# Quantitative analysis of *in-vivo* microbubble distribution in the human brain

**DOI:** 10.1038/s41598-021-91252-w

**Published:** 2021-06-03

**Authors:** Francesco Prada, Antonio G. Gennari, Ian M. Linville, Michael E. Mutersbaugh, Zhihang Chen, Natasha Sheybani, Francesco DiMeco, Frederic Padilla, John A. Hossack

**Affiliations:** 1 Department of Neurological Surgery, University of Virginia Health System, Charlottesville, VA USA; 2 Department of Neurosurgery, Fondazione IRCCS Istituto Neurologico C. Besta, Milan, Italy; 3 Focused Ultrasound Foundation, Charlottesville, VA USA; 4 Neuroradiology Unit, Fondazione IRCCS Istituto Neurologico C. Besta, Milan, Italy; 5 Department of Radiology, Cattinara Hospital, University of Trieste, Trieste, Italy; 6 Biomedical Engineering, University of Virginia, Charlottesville, VA USA; 7 Department of Pathophysiology and Transplantation, University of Milan, Milan, Italy; 8 Department of Neurological Surgery, Johns Hopkins Medical School, Baltimore, MD USA; 9 Department of Radiology, University of Virginia Health System, Charlottesville, VA USA; 10 Ultrasound NeuroImaging and Therapy Lab, Fondazione IRCCS Istituto Neurologico C. Besta, Via Celoria 11, 20133 Milan, Italy

**Keywords:** Blood-brain barrier, Neuro-vascular interactions, Cerebrovascular disorders, CNS cancer, Biomedical engineering, Surgical oncology, Image processing, Neurosurgery

## Abstract

Microbubbles (MB) are widely used as contrast agents to perform contrast-enhanced ultrasound (CEUS) imaging and as acoustic amplifiers of mechanical bioeffects incited by therapeutic-level ultrasound. The distribution of MBs in the brain is not yet fully understood, thereby limiting intra-operative CEUS guidance or MB-based FUS treatments. In this paper we describe a robust platform for quantification of MB distribution in the human brain, allowing to quantitatively discriminate between tumoral and normal brain tissues and we provide new information regarding real-time cerebral MBs distribution. Intraoperative CEUS imaging was performed during surgical tumor resection using an ultrasound machine (MyLab Twice, Esaote, Italy) equipped with a multifrequency (3–11 MHz) linear array probe (LA332) and a specific low mechanical index (MI < 0.4) CEUS algorithm (CnTi, Esaote, Italy; section thickness, 0.245 cm) for non-destructive continuous MBs imaging. CEUS acquisition is started by enabling the CnTI PEN-M algorithm automatically setting the MI at 0.4 with a center frequency of 2.94 MHz–10 Hz frame rate at 80 mm—allowing for continuous non-destructive MBs imaging. 19 ultrasound image sets of adequate length were selected and retrospectively analyzed using a custom image processing software for quantitative analysis of echo power. Regions of interest (ROIs) were drawn on key structures (artery–tumor–white matter) by a blinded neurosurgeon, following which peak enhancement and time intensity curves (TICs) were quantified. CEUS images revealed clear qualitative differences in MB distribution: arteries showed the earliest and highest enhancement among all structures, followed by tumor and white matter regions, respectively. The custom software built for quantitative analysis effectively captured these differences. Quantified peak intensities showed regions containing artery, tumor or white matter structures having an average MB intensity of 0.584, 0.436 and 0.175 units, respectively. Moreover, the normalized area under TICs revealed the time of flight for MB to be significantly lower in brain tissue as compared with tumor tissue. Significant heterogeneities in TICs were also observed within different regions of the same brain lesion. In this study, we provide the most comprehensive strategy for accurate quantitative analysis of MBs distribution in the human brain by means of CEUS intraoperative imaging. Furthermore our results demonstrate that CEUS imaging quantitative analysis enables discernment between different types of brain tumors as well as regions and structures within the brain. Similar considerations will be important for the planning and implementation of MB-based imaging or treatments in the future.

## Introduction

Microbubbles (MBs) are a widely used ultrasound contrast agent (UCA). They comprise an external lipid, protein, or polymer shell encasing a gaseous core, possessing a nominal diameter in the range of 1–4 microns. An individual microbubble response to ultrasound insonation is a function of insonating frequency, pulse amplitude and shape, and microbubble mechanical resonant frequency. Additionally, there is a strong nonlinear response that gives rise to a range of sub and super harmonics. These harmonic signals allow for high-performance separation of microbubble origin signal from adjacent tissue signal, which only produces a relatively weak harmonic signal. These unique qualities may be used in both imaging and therapy. In contrast-enhanced ultrasound (CEUS), MB origin signals are distinguished using harmonic specific ultrasound imaging sequences, allowing excellent vessel visualization and unique tissue perfusion evaluation for diagnostic purposes^1^. On the contrary, when combined with focused ultrasound (FUS), MBs enable various biological effects, such as the safe and reversible opening of the blood–brain barrier (BBB), easing drug delivery^2^, or tissue damage, in case of inertial cavitation^3–6^.

CEUS has already helped to distinguish and characterize different entities affecting various organs^7,8^. Recently, MB imaging has been implemented in neurosurgical procedures on a large scale, taking advantage of the craniotomic access, to image several different brain pathologies^1^. However, the main goal in this approach was to derive real-time information to tailor the surgical treatment to the patient’s need. To date, most of the studies on CEUS in brain lesions have used qualitative (visual assessment) or semi-quantitative analysis only, and only rare attention has been directed towards the distribution and localization of MBs in different areas of the normal human brain^9–11^. Thus far, analysis has only been attempted with transcranial sonography^12^. This lack of quantification limits the guidance of CEUS surgery or MB-based FUS treatments. Indeed, the distribution of MBs in the brain is not yet fully understood, with areas such as the basal ganglia showing an enhancement similar to the ones of high-grade gliomas. Furthermore, MB targeted FUS is actually performed assuming a normal distribution of MBs in the brain^13^, ultimately leading to potential tissue damage outside the intended target. Since MBs are a strictly intravascular contrast agent, each pixel in CEUS images provides a representation of vascular replenishment rate, and, therefore, blood flow. Quantitative analysis is objectively superior to qualitative analysis^14^, enhancing the understanding of MB dynamics and distribution in the human brain enabling safer treatments, involving both surgery and FUS, by increasing tissue recognition and, logically, will reduce complications. Additionally, gaining insight into brain vascular regulation will be a cornerstone to facilitate further studies on CEUS guided surgery and FUS treatments.

The objective of this paper is to show that intraoperative quantification of MBs distribution in different regions of the brain is feasible and that this quantification may help to discriminate between tumoral and normal brain tissues, and aims to introduce new information relating to real-time brain vascularization.

## Materials and methods

### Study design

The Institutional Review Board of the Fondazione IRCCS C. Besta (Milan, Italy) approved the use of CEUS as part of a standard procedure for neurosurgical guidance. Therefore, in our study, based on routinely collected anonymized data from patients who underwent neurosurgical procedures between June 2013 and July 2018, a specific informed consent was waived by the Institutional Review Board of the Fondazione IRCCS C. Besta (Milan, Italy). As part of a Biomedical Engineering Capstone Project at the University of Virginia (Charlottesville, USA) we retrospectively evaluated the images of twenty-one patients operated at the Fondazione IRCCS C. Besta for brain tumor removal with ultrasound guidance where CEUS imaging was of adequate length (> 40 s). Successively, an off-line quantitative analysis was performed using an in-house developed image processing software for MB signal analysis. All methods were carried out in accordance with relevant guidelines and regulations.

### Intraoperative US examination—CEUS

We used an ultrasound machine (MyLab Twice, Esaote, Italy) equipped with a multifrequency (3–11 MHz) linear array probe (LA332) and a specific low mechanical index (MI < 0.4) CEUS algorithm (CnTi, Esaote, Italy; section thickness, 0.245 cm) for non-destructive continuous MBs imaging. Ultrasound evaluation was performed placing the probe directly on the intact dura mater, through the craniotomy window. Standard B-mode imaging was performed in two orthogonal planes to identify the lesion, US landmarks and neighboring structures. The field of view (FOV) was adjusted in order to encompass as many structures as possible. The probe was then placed on the more significant section of the surgical field. The ultrasound focus, dynamic range, frames per second analysis, echo-signal gain and signal persistence were adjusted before CEUS evaluation. CEUS acquisition was started by enabling the CnTI PEN-M algorithm automatically setting the MI at 0.4 with a center frequency of 2.94 MHz–10 Hz frame rate at 80 mm—allowing for continuous non-destructive MBs imaging.

A single bolus (2.4 ml [5 mg/ml]) of sulphur hexafluoride–filled lipidic MBs, (SonoVue, Bracco, Milan, Italy) was injected in a peripheral vein, followed by a 10-mL saline flush, in each patient. Synchronously with injection, a timer and cine clip were started to study the MBs kinetics in the lesion from time of arrival in major vessels to washout. For quantitative analysis purposes, we selected only image clips in which the probe was kept still for at least 40 s (average: 45″, range 35″–300″) since MB arrival.

### ROI selection and data processing

Acquired cine clips were analyzed using a dedicated, newly developed, software created with MATLAB’s App Designer in edition R2019a. Each clip was loaded and displayed. Prior to image quantification, CEUS sequences were converted to sets of 8-bit greyscale images. An experienced neurosurgeon (FP) drew three different regions of interest (ROI), selecting between several shapes (circular, rectangular, or freehand drawing), one ROI on an artery, one ROI in the tumor and one ROI in the white matter (WM) structures. Whenever other diagnostically significant regions (such as grey matter, basal ganglia, corpus callosum) were imaged, adjunctive ROIs were drawn to analyze these specific areas. Pixel intensity values within the ROIs were calculated for each frame of the clip and to generate the time-intensity curve (TIC). In necrotic tumors, or if the tumor was too large to entirely fit in the FOV, the tumor ROI was only drawn on a section rather than on the whole tumor. In those cases, we compared the most vital part of the neoplasm with the parenchyma and vessels. Two summary measures were derived in this analysis. The peak enhancement (PE) was calculated as the intensity maximum value for each ROI. The area under the curve (AUC) was derived as the integral of the TIC. For the normalization and for each patient, the AUC for brain and tumor tissues was obtained as the ratio of their AUC to the AUC of the artery, in order to compensate for discrepancies that could arise due to the different acquisition durations between patients. All ROIs, TICs, and computed metrics were saved and could be recalled through the graphical user interface. The software also allowed for thresholding, in order to define lower and upper values of intensity, as a rapid way to highlight prominent areas of interest, or identify any notable physiological patterns. If a pixel grey level exceeded the threshold, the pixel grey level was rounded up to white (8 bit all ones: 255). This is approach is useful as a quick way to highlight prominent areas of interest, or identify any notable physiological patterns.

### Statistical analysis

For each patient, paired t-tests between the different tissue types were performed for both the peak intensity value and the normalized AUC. All 19 data sets were used to test any existing differences between normal brain tissue and artery. Due to an acquisition related issues (artifacts), one data set had to be removed, when comparing the difference between tumor and normal brain or artery.

## Results

### Qualitative and quantitative analysis

CEUS images from twenty-one patients (10 males, 11 females; mean age at surgery 49 years old, range 19–71 years) with primary brain neoplasm were evaluated; tumors histotypes are summarized in Table 1.

**Table 1:** Patients description including tumors histotypes.

N	Sex	Age	Histo	Site
1	M	58	Glioblastoma	Parietal
2	F	21	Glioblastoma	Temporal
3	M	38	Anaplastic Glioma	Insular
4	F	65	Glioblastoma	Frontal
5	F	68	Meningioma	Clinoid
6	F	49	Recurrent Glioblastoma	Temporal
7	M	68	Metastasis	Frontal
8	F	19	Glioblastoma	Frontal
9	F	62	Glioblastoma	Parietal
10	F	71	Anaplastic Glioma	Parietal
11	M	29	Glioblastoma	Thalamic
12	F	34	Glioblastoma	Temporal
13	F	43	Meningioma	Clinoid
14	M	54	Cortical Dysplasia	Frontal
15	M	58	Metastasis	Parietal
16	M	69	Glioblastoma	Parietal
17	F	67	Glioblastoma	Parietal
18	F	27	Glioblastoma	Temporal
19	M	59	Meningioma	Clinoid
20	M	65	Glioblastoma	Frontal
21	M	55	Anaplastic Glioma	Parietal

**Figure 1:**
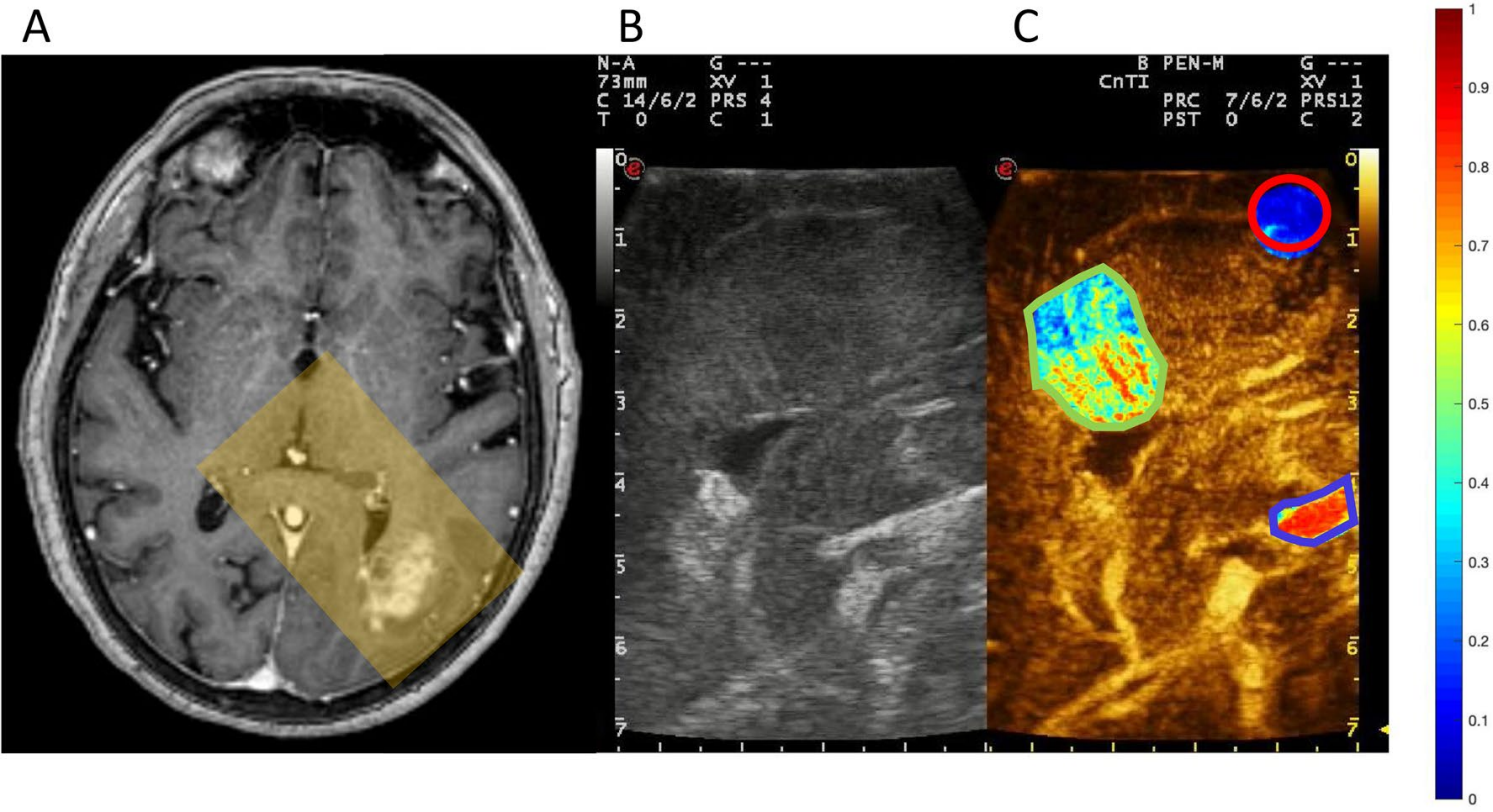
Qualitative view of microbubble density in selected regions in a case of parietal glioblastoma: (**A**) Gd-enhanced T1 axial MRI showing the lesion. A transparent yellow rectangle shows the plan of insonation through the craniotomy, corresponding to the US images on the right. Screenshot of the US monitor showing low mechanical index B-mode (**B**) and CEUS images (**C**). In (**C**) three ROIs are depicted, circled in blue (artery), green (tumor) and red (white matter), displaying MB density in JET colorimetric scale on the side.

All of the twenty-one acquisitions recorded were successfully analyzed with the in-house designed software, to draw ROIs on selected areas (Fig. 1) and to process the data to obtain the corresponding TIC. However, two data sets were contaminated with motion artifacts that arose during image acquisition and were discarded without further analysis. To avoid such problems refined acquisition protocols are under development.

**Figure 2:**
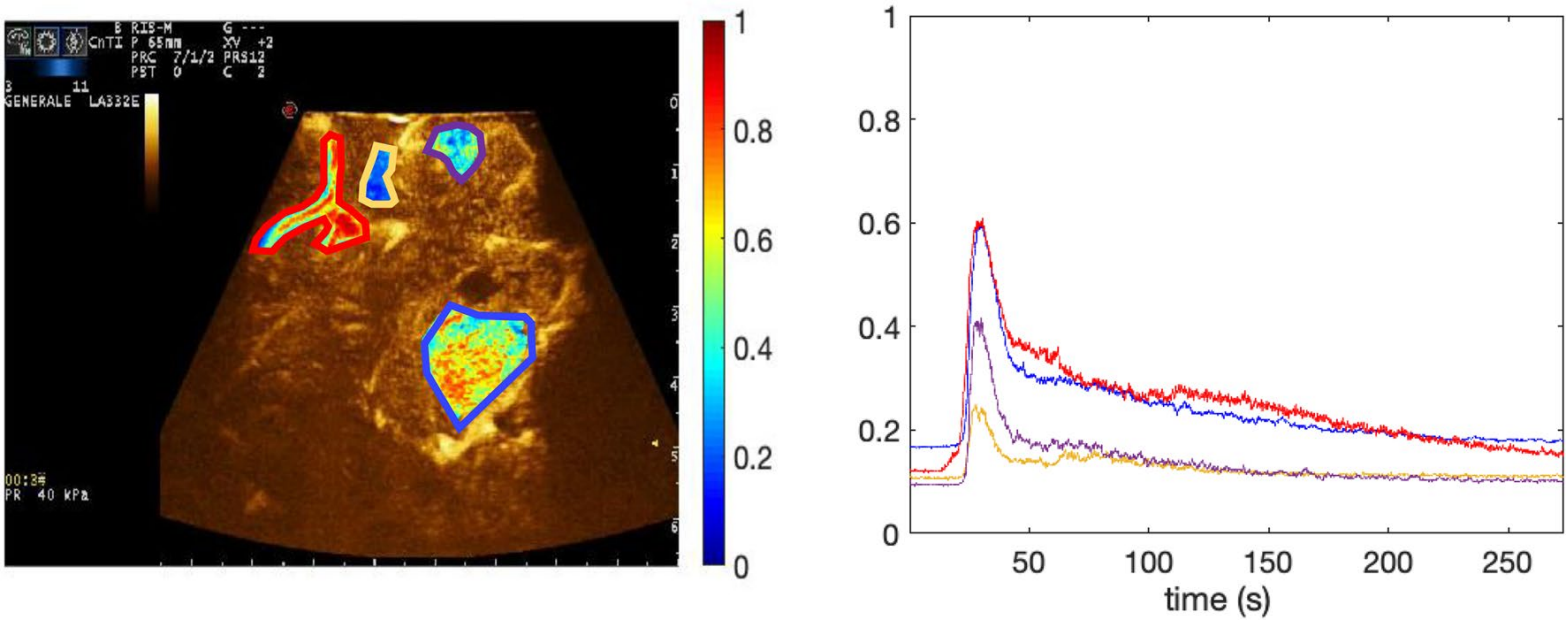
Time Intensity curves to show the microbubble distribution of distinct brain structures over time. Displays the extracted intensity values from each ROI over the microbubble life cycle in a patient diagnosed with a mesial temporal lobe high grade glioma. The quantified time-intensity curves show each selected structure of the brain over 1696 frames sampled at 20 Hz, approximately an 85 s timespan. The x-axis displays time in seconds over the duration of the 85 s video. Each intensity value is equivalent to the average intensity of a given ROI for each frame, or in time the average of every 0.05 s. Qualitatively, the four ROI’s show differences in distribution, with the artery and tumor structures showing the higher microbubble density, compared to white and grey matter.

**Figure 3:**
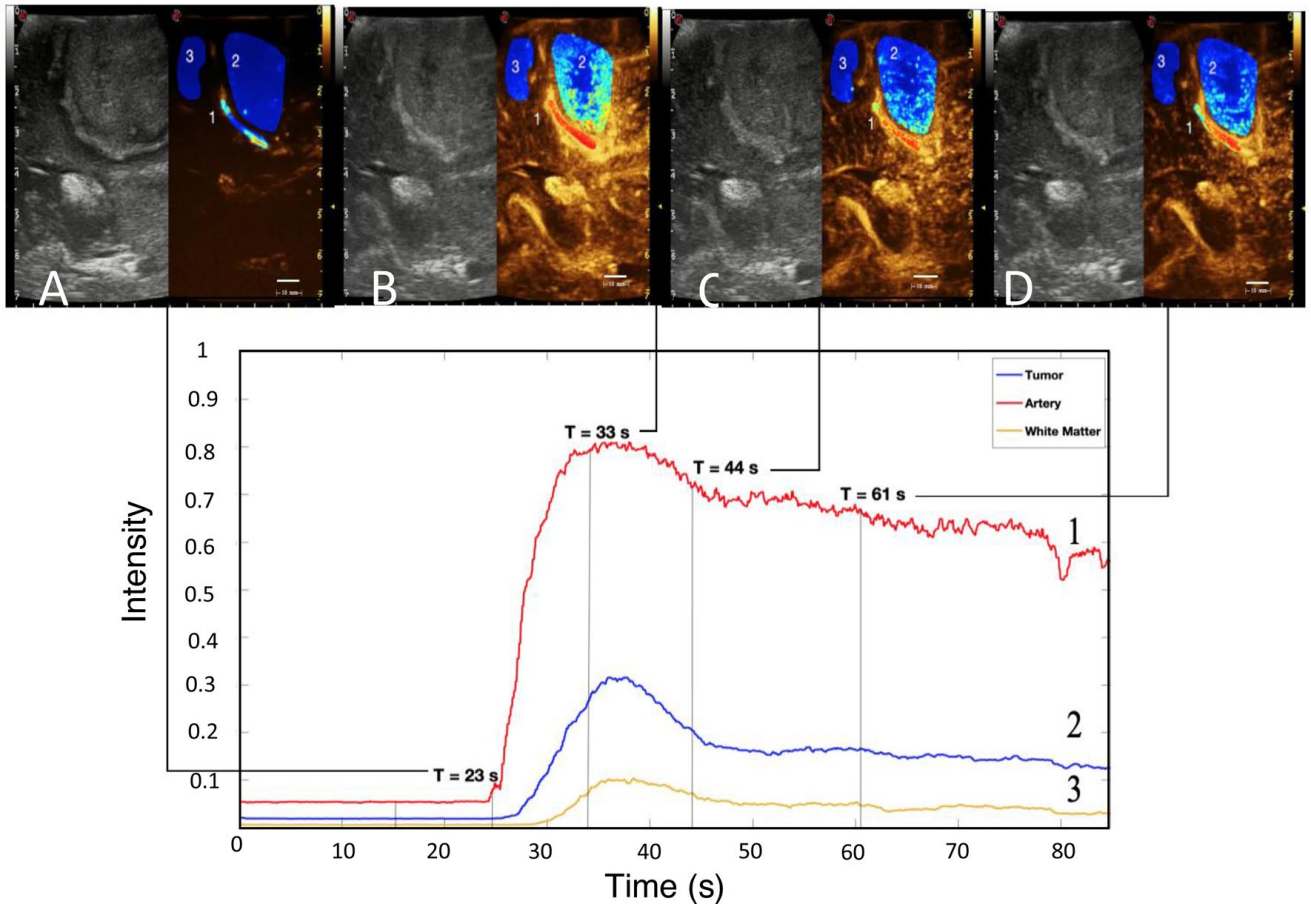
Time intensity curve correlation with phases of the microbubble life cycle, showing different amount of microbubble in the same area. (*Artery (red-1), Tumor (blue-2), White Matter (yellow-3)*) In the upper panel 4 screenshot with low-MI B-mode and harmonic imaging in a case of parietal glioma: pre-injection microbubble concentration is at a baseline at 18 s and at 23 s the concentration starts to rise in the artery post-injection (**A**). The second time-point represents the beginning of the wash-in phase post-injection. The intensity is greatest between 33 (peak—**B**) and 40 s (early wash out—**C**) and has started to wash-out by 44 s. The late wash-out phase at 61 s (**D**) shows a still dense distribution of microbubbles in the artery, while the white matter has returned to approximately baseline.

From a qualitative standpoint, there were notable differences in the distribution of MBs between three main regions across all patients: arteries, tumor and brain parenchyma (Fig. 2). In CEUS images, the artery had the highest enhancement among all structures, followed by the tumor and brain regions, respectively. Tumor heterogeneous structure, composed of a mixture of vital and necrotic areas, especially in high-grade glioma (HGG), could explain its intermediate enhancement. The brain ROIs showed little MBs perfusion through time, demonstrating minimal intensity increase with time. Continuous evaluation helped define different brain CEUS enhancement phases. The arterial phase, in which only arterial structures are visible, started between 20 and 30 s after MB injection and endured for approximately 6–8 s. Major arteries enhanced first vividly, followed by sulcal arterioles. Successively, approximately 7–9 s after MBs arrival, we imaged a faint enhancement of cortical grey matter as well as of the basal ganglia, which lasted until 20 s. Brain parenchyma enhancement grossly demonstrated a dual enhancement: cortical layers showed a brighter enhancement, along with the basal ganglia, while on the other hand WM had a longer and fainter enhancement. Contemporary (9–11 s) peripheral, subcortical veins started draining MBs into deep venous structures (14–16 s). White matter enhancement lasted a little longer than that of grey matter (25 s) (Fig. 3). Venous enhancement was less conspicuous than the arterial one, starting at 5–10 s after MB arrival. Grey matter was not clearly visible and measurable in all cases, because of its size, shape and vicinity to the probe at the time of acquisition.

The recorded TICs in the brain presented shapes similar to the one already measured with CEUS imaging in other organs^15^.

In all cases and structures analyzed, five-phases of MB dynamics were measured: baseline, wash-in phase, peak enhancement, early wash-out phase, and late wash-out phase (Fig. 3). Pre-injection the density of MBs is absent, but it quickly starts to rise once MBs are injected. After the peak enhancement is reached it begins to slowly wash-out, returning towards the baseline.

**Figure 4:**
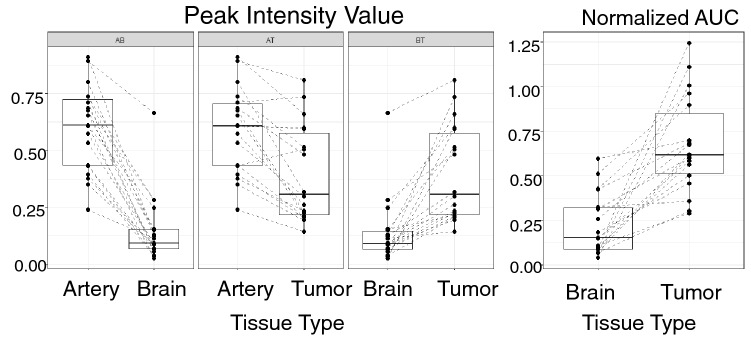
Boxplot for Peak Intensity and for Normalized AUC of brain and tumor with paired observations (for each patient) connected by dashed lines. AB: Artery v. Brain; AT: Artery v. Tumor, BT: Brain v. Tumor.

Analysis of the brain parenchyma using three different ROIs allowed the quantification of the differences already qualitatively observed between the various tissue types. Analysis of PE and AUC values was performed in 19/21 (90.5%) data sets. Out of all nineteen samples, WM structures had the lowest average MB intensity at 0.175 units, followed by tumors at 0.436 units, with artery samples having the highest intensity with an average of 0.584 arbitrary units (Table 2, Fig. 4). One dataset (patient 11), presented outlier values of WM PE but had similar abnormally high values also in the tumor and artery ROIs.

**Table 2:** Summary table of peak intensity values in the nineteen samples analyzed.

Groups	Count	Sum	Average	Variance
White matter	19	3.329	0.175	0.034
Tumor	19	8.284	0.436	0.046
Artery	19	11.091	0.584	0.048

The results from the paired t-tests showed that, under a 0.05 confidence level, all comparisons tested show significant differences in both peak intensity value and normalized approximate AUC (Table 3). For the peak intensity, values were found to be lower for brain than for the tumor and the artery, and lower for the tumor than for the artery (Fig. 4, Tables 2 and 3). Similarly, the normalized AUC was found to be lower in the brain than in tumors (Tables 2 and 3).

**Table 3:** *p* value table for all paired t-tests, for both peak intensity and normalized AUC.

	Brain and tumor	Artery and brain	Artery and tumor
Peak intensity value	1.785e−05	8.421e−10	1.1084e−4
Normalized AUC	1.009e−05	NA	NA

### Illustrative cases

#### Timing differences

**Figure 5:**
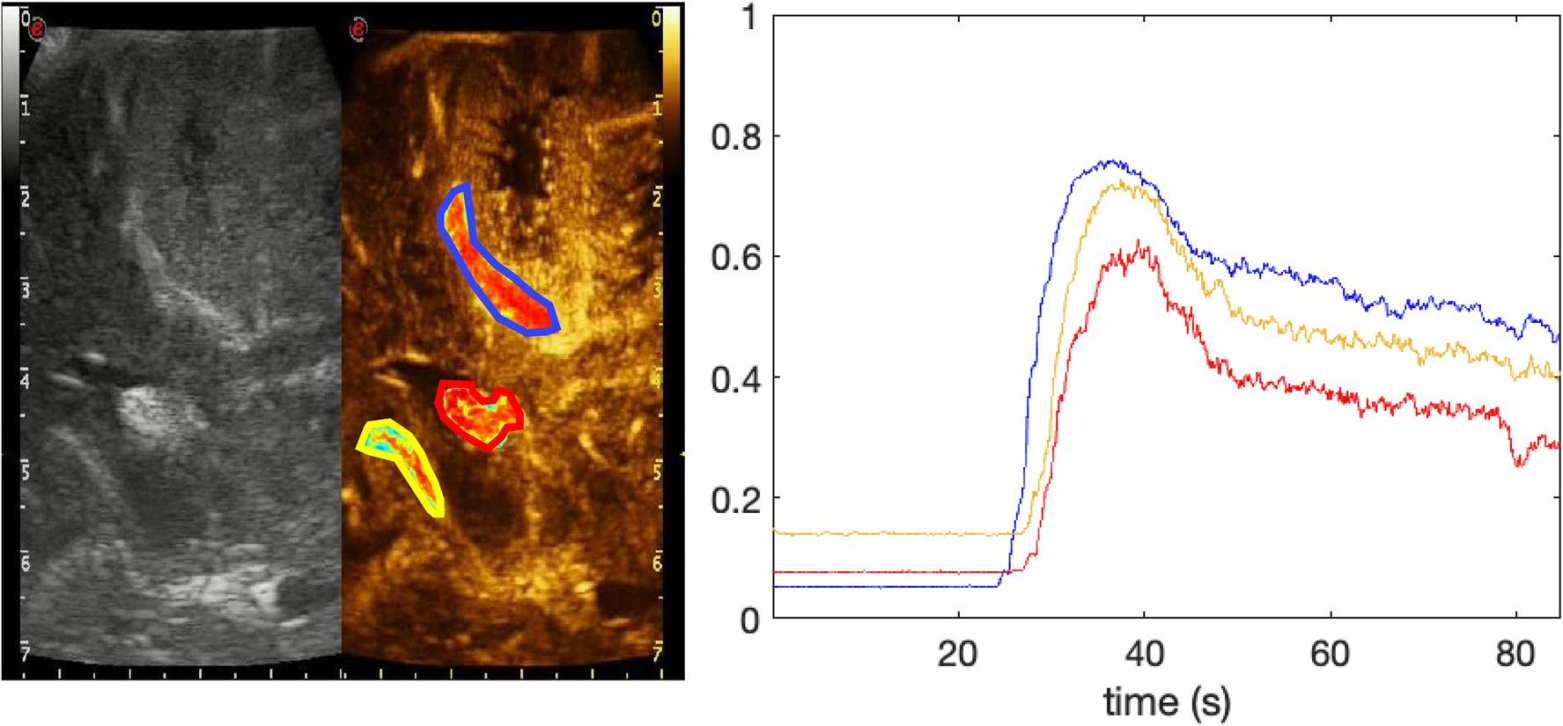
Timing differences between different vascular and cerebral structures. Left panel: B-mode and CEUS images on a still frame. The 3 regions of interests selected using the analysis software are displayed in color on the CEUS image using colorimetric scale for MBs concentration and circled according to the plot—sulcal artery, blue—choroid plexus, red—basal vein, yellow. Right panel: Time-intensity curve plot. Continuous evaluation allowed the detection of MBs arriving in a sulcal peritumoral artery following an orthograde flux, in one of the deep basal veins and successively in a highly vascularized structure such as the choroid plexus. The plotted graphs show this difference as a delayed shift in the PE of the different structures with a delay of 1 s from each one as seen on the upper scale.

Continous evaluation allowed the detection of MBs arriving in a sulcal peritumoral artery (20–30 s after injection), following an orthograde flux, and successively in the deep basal veins (5 s after MB’s arrival in the arterial bed. The plotted graphs show this difference as a delayed shift in the PE of the venous structure (Fig. 5).

#### Intensity differences

**Figure 6:**
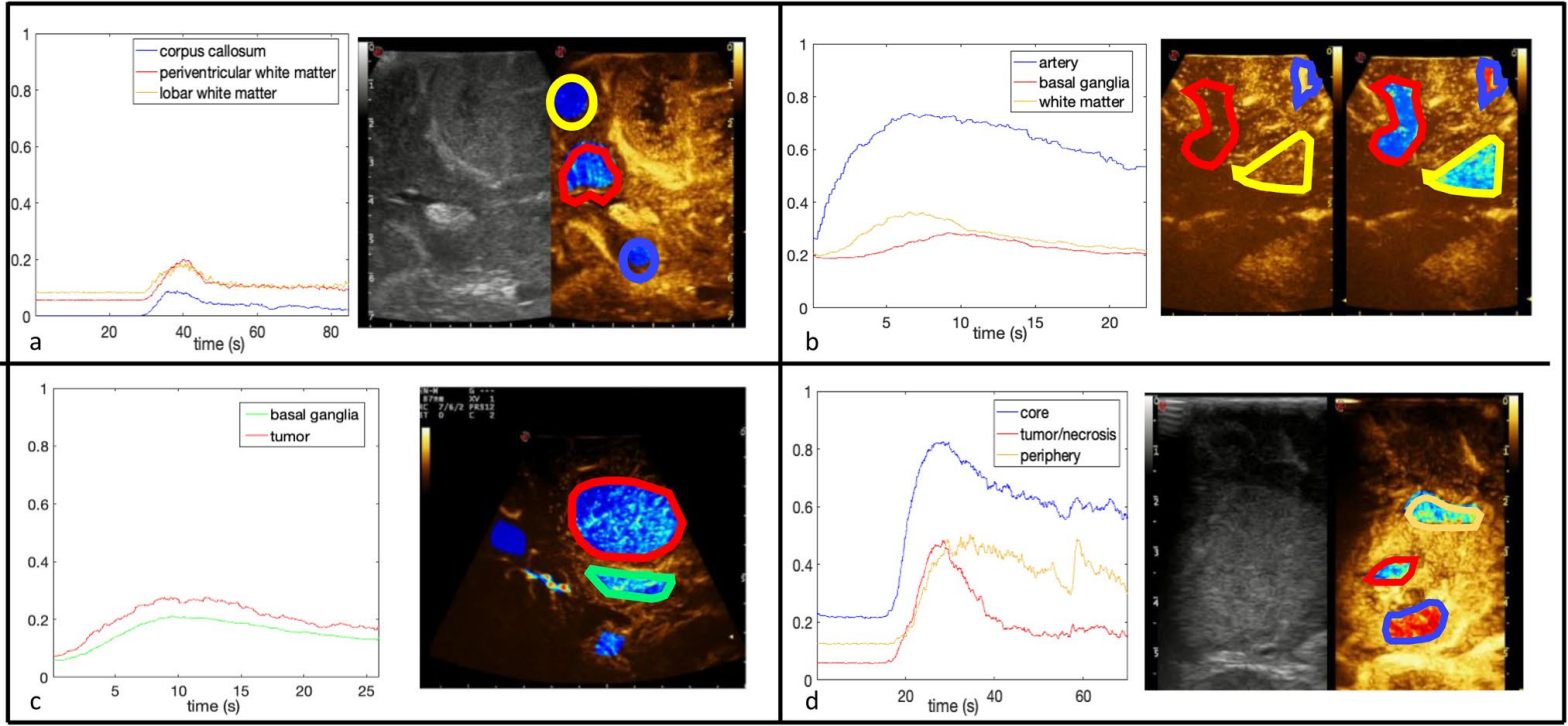
Differences between cerebral structure—particular cases, see text. (**a**) white matter structures; (**b**) parenchymal structures; (**c**) parenchyma and tumor; (**d**) intra-tumoral areas (x axis: frames ; y axis: intensity).

Our quantitative analysis software also was able to show, in the appropriate cases, differences between: (a) WM and grey matter structures—(b) WM structures—(c) differences between same tumor areas—(d) absence of differences between certain brain parenchyma structures and brain tumors (Fig. 6). These differences are probably related to the vessel density in different areas as well to the blood flow; this is directly related to the different amount of MBs observed during CEUS evaluation, as the MB presence in a certain area is a stochastic phenomenon related to those factors.

- Differences between different WM areas
  Surprisingly, we found intensity differences even within different white matter areas: the corpus callosum, as well as other compact WM bundles, appeared to have a slightly lower enhancement compared to other areas (Fig. 6a). Periventricular WM showed the higher MBs concentration, probably because of its role as a collector of the deep venous blood flow: the abundant presence of medullary veins, getting progressively closer toward the ventricle, is probably responsible for this finding.
- White matter versus Basal ganglia
  In one case, we found a striking difference in terms of quantitative perfusion between lobar WM compared to basal ganglia. As visible in the coronal section showed in Fig. 6b, there is a clear difference between the thalamus/putamen ROI, the lobar WM ROI, and the grey matter superficial ROI.
- Absence of differences between tumor/brain
  Despite a consistent difference in enhancement (and perfusion) between the tumor and the WM parenchyma in all cases, we found a single case (a diffuse astrocytoma, World Health Organization 2016 grade III) in which a similar pattern of enhancement on the quantitative analysis between the main lesion and the basal ganglia was found (Fig. 6c). As highlighted above, the basal ganglia have indeed a higher amount of microvessels compared to the lobar parenchyma, therefore they show a perfusion pattern with higher PE and steeper time to peak rise, superimposable to that of mildly enhancing tumors. This should be kept in mind when using CEUS as surgical guidance in case of deep-seated lesions or when dealing with MBs mediated US treatments.
- Intra-tumoral analysis
  Large differences in terms of TIC can be found instead within the same lesion, as shown in a case of a frontal glioblastoma (GBM), where the ROIs applied to the necrotic core and two different peripheral areas show a different perfusion pattern (Fig. 6d). As mentioned above the same concept needs to be applied when planning MBs mediated treatments on brain tumors or when performing surgical resection with CEUS guidance.

## Discussion

Our study describes the first large-scale implementation of quantitatively analyzed CEUS intraoperative images in order to evaluate the different degrees of perfusion of brain tumors and brain areas. This quantification allowed comparisons between different regions of the brain, such as the white and grey matter regions, and different structures, such as the comparison between and within tumors. Analyzed anatomical structures (artery, brain parenchyma, tumor) were visible in all cases with CEUS; for each structure, a quantitative analysis was performed obtaining TIC. The shape and morphology of the obtained TIC were reproducible consistently for each structure in all cases, with arteries showing the highest degree of enhancement, followed by tumor and brain parenchyma.

As a matter of fact, CEUS quantitative analysis provides pivotal information regarding MB distribution and tissue perfusion in any organ^16,17^ and its application to direct cerebral CEUS imaging will pave the way to further understanding of this organ physiopathology. Few reports have shown the feasibility of transcranial CEUS quantitative analysis, whereas only one study was performed intra-operatively. The formers were obviously hampered by the presence of the skull, limiting the visualization options to the temporal window, dramatically reducing imaging resolution. Contrary, the study by Wang et al., representing the sole conducted intraoperatively on a large cohort of patients, was flawed by many problems, from acquisition to CEUS analysis^12,18,19^.

In our paper, we provide the first extensive quantitative analysis of cerebral MB distribution.

### Enhancement phases

Setting the probe stationary on the dura allowed us to differentiate different MB enhancement phases of brain parenchyma. This simple, but frequently underrated analysis, is mandatory to define the most appropriate time delay (enhancement phase) after MBs injection to maximize the conspicuity of detectability of a specific pathology^20^. Although, wider cohorts are needed, in our small sample study we were able to identify various brain enhancement phases. MB intravenous administration can be performed using either bolus or extended infusion techniques with each providing information on tissue perfusion. Bolus injection was used in all our cases. In bolus injections, an operator injects the MBs manually in a peripheral vein, followed by a saline flush. The MBs will therefore arrive through the arterial systemic blood supply with a delay of about 10–15 s post-injection. For extended infusion, an automatic power injector system is used, providing a more homogenous MBs concentration between the different tissue types^21^. This latter approach is however more complex and may not be routinely used in intraoperative CEUS, whereas bolus injection is easier to implement in an operation room setting. Infusion, however, could be more suitable for the therapeutic use of MBs, such as BBB opening, where a homogenous concentration of MBs will be beneficial. When using the disruption-replenishment technique (switching from low mechanical index to the highest possible, generating MBs disruption, and then returning to the previous image setting), the acquisition can be repeated few times during an infusion, at different planes, and can possibly improve the accuracy of the measurements.

Since many patients’ issues such as cardiac output, hydration status or body temperature could slightly modify the time MBs take to reach the brain, we selected to start counting distinct enhancement phases when the first CEUS signal was detectable. Indeed, in a radiologic lexicon, the viable parts of HGGs tend to have a rapid and intense wash-in during the arterioles phase, which endures over the late WM phase. Having defined such temporal distinction could help to better understand the pathological changes induced by tumor masses in the brain such as venous engorgement, or neoangiogenesis, and to better plan surgical strategies and MB mediated treatments.

### Cerebral structures enhancement

As expected, due to its intrinsic histology and vascularization, WM exhibited significantly lower MB density compared to arteries and tumors. The reduced vascular caliber and low flow generated fainter peak enhancement, but CEUS high spatial and contrast resolution enabled superficial imaging and deep medullary veins as well as anastomotic veins and trans-cerebral ones. Furthermore, differences within WM enhancement were observed. Periventricular zones had a higher enhancement compared to deep-seated, thick, commissural fibers such as the corpus callosum. This could be related to the venous anatomy of periventricular WM with its abundant presence of medullary veins, getting progressively closer toward the ventricle^22^. In any event, lower enhancement generated lower inter-patients data variance compared to arteries and tumors (Table 1). Therefore, the WM could be a good internal reference to be compared with other brain structure measures. As previously discussed, many factors could determine slight variation in tissues enhancement. Standardizing measures to a reference area could help reduce inter-patient and inter-group results differences. Additionally, a similar approach is routinely used in the quantification of computed tomography (CT) or MR perfusion images^23^..

### Quantitative versus qualitative implications

Many of the current imaging modalities for the brain lack fully implemented quantification methods, leaving much of the data to be qualitatively assessed. This leads to challenges in the treatment of patients since the absence of quantified data limits our confidence in their prognosis. For example, treatment of brain tumors presents challenging problems due to the varying tumor types^24^. Moreover, quantitative data allows for a better understanding of the pathophysiology of angiogenesis of benign and malignant neoplasia and it enables comparison between imaging techniques.

Eyding et al. already proved the ability of quantitative CEUS evaluation comparison with CT or MR perfusion imaging in a cohort of patients with acute anterior circulation occlusive stroke^23^. Contrary to our study, the authors used the temporal bone as an acoustic window to image MB brain distribution. Since they used a contralateral and an ipsilateral approach (proving the former to be better than the latter) a low-frequency probe usage was essential. However, lower ultrasound frequencies limit spatial resolution, so the anatomical detail and the capability to distinguish between different brain structures of their images are not comparable to ours. Furthermore, the use of the temporal bone window does not allow scanning with precision areas such as the vertex or the parietal and occipital lobes. In our opinion, the complex architecture of brain parenchyma demands higher image details to fully explore quantitative CEUS potentials, as it is possible to obtain through a craniotomic window. Indeed they performed a more accurate analysis of TICs as it is usually performed in standard diagnostic settings, evaluating numerous CEUS quantification parameters such as *appearance time of the bolus in your ROIs, time to peak enhancement, wash-in, and washout time*^25^*.* However, in our report, we used two parameters only, namely PE and AUC for a different reason: relying on previous experiences from our group (1, 9–13) PE and AUC were the most important parameters to be evaluated intra-operatively for surgical guidance, with qualitative and semi-quantitative analysis, visually providing the highest degree of differentiation between different structures.

Direct CEUS imaging with quantitative analysis was performed by Wang J. et al. in a series of 49 patients^18^. Despite being the first known report in which quantitative analysis was attempted it is flawed by different issues, as it is not clear in how many cases quantitative analysis was performed. In their TICs many peaks are visible, probably due to probe movements, essentially sliding the ROI from an area to another, completely changing the waveform, or else due to several bolus injections. Furthermore, parameters on the X and Y-axis of the TIC were not specified, nor it is clear if they normalized their curves across different patients^18^.

In our series, we managed to obtain homogenous data across all patients, allowing to perform a thorough statistical analysis. The statistically significant differences observed between the different brain areas, for both peak enhancement and AUC, suggest that those two measures could be predictive variables in discriminating different types of tissue in the brain. Considering that the aim of our paper is to demonstrate that different cerebral structures have different concentrations of MBs in time, we chose the most simple parameters that were able to provide this type of information, namely PE and AUC. Indeed, other parameters can provide valuable information, and further and more accurate studies are warranted. Technical constraints also dictated our choice as in some cases, it was difficult to clearly define a baseline intensity level because the data recording was not started early enough during the acquisition in the operative room, preventing a normalization and/or computation of some parameters such as time to peak or wash-in time. We definitively plan to improve our data acquisition settings in order to implement our comprehension of MBs kinetic in the brain. We also believe that a classifier to predict different tissue types based on summary measures such as AUC and peak density value can be derived in future studies on a larger cohort of patients.

Our study also bears important and provocative implications for the field of therapeutic ultrasound. Herein, we demonstrate the feasibility of intraoperative MBs quantification in different regions of the brain. Furthermore, we generate evidence for the differential accumulation and retention of MBs across normal brain structures as well as in the malignant setting. Focused ultrasound (FUS)-mediated disruption of the BBB is an example of a therapeutic strategy that could be influenced by such findings, given that this application relies on the presence of intravascular MBs. FUS BBB opening is rapidly advancing as a non-invasive, safe, and repeatable strategy^26^ to treat a wide variety of neurological pathologies^25–38^.

Given the critical role that MBs play in the BBB opening response and its temporary nature, efforts have been placed in the development of passive cavitation detection (PCD) systems for monitoring and adjusting parameters on the basis of acoustic emissions^29,39,40^. However, acoustic feedback from these systems is not currently optimized on the basis of differential spatial accumulation or flow of intravascular MBs across brain regions and structures. Moreover, since the composition, size, and dose of MBs all play a known role in the bioeffects associated with BBB opening, the accumulation of MBs within various structures of the CNS certainly does so as well^41–44^ as it has been shown in WM versus grey matter owing to different vascularity and resultantly different MB concentration^5^.

Clinically, FUS BBB opening has already been demonstrated safe and feasible in the context of GBM^6,41,45^, ALS^42^, and Alzheimer’s disease^43^. However, in these pathologies is underpinned by what we demonstrate herein to be noteworthy differences in MB distribution based on cerebral and/or tumor vascularity. This consideration is paramount to the effective “response” of patients to FUS for BBB opening, even within the category of brain malignancies alone.

### Limitations and future work

There are some limitations to this research that could be addressed in future studies. First, when comparing the same structures between different individuals there is an assumption that their blood flow dynamics and structural area are similar. This was particularly difficult to address due to the small sample size of this study. Out of twenty-one patient samples, nineteen were successfully analyzed, and the conclusions of our study will now need to be reproduced in a larger cohort. Secondly, its retrospective design limits the possibilities to standardize all the CEUS acquisition settings which, if similar and optimized would result in more consistent and higher quality data. Thirdly, more detailed analysis and imaging compensation must be carefully considered and improved: ultrasound attenuation should be taken into account and compensated for using an improved software. However, in our experience, MBs “brightness” reflects the anatomical microvasculature of the cerebral structures under examination. We also limited attenuation using a FOV depth never larger than 5 cm, and we took care to avoid placing ROI below a highly vascularized area and tried to place ROIs at the same depth. However, in order to select three different anatomical regions (tumor–artery–brain) consistently in all cases, it was not always possible to select ROIs at the same depth. We also thoroughly checked all cine clips before analysing them, searching for pseudoenhancement, which could alter our evaluation excluding the one flawed by artefacts^22^. It has to be said that far-wall pseudoenhancement has only been detected with large-calibre vessels, which are not present in the brain. A better compensation could reinforce the differences found between arteries, brain, and tumors, but would not change the conclusion of our analysis.

MBs lifespan was not evaluated as this was not the aim of this work: for our purpose it was sufficient to evaluate the wash-in/peak of enhancement/wash-out phases. Furthermore, being an intra-operative setting, it was of no value for the surgical strategy to prolong the observation until MBs exhaustion. From other observed cases MBs can be detected up to 5 min after injection.

**Figure 7:**
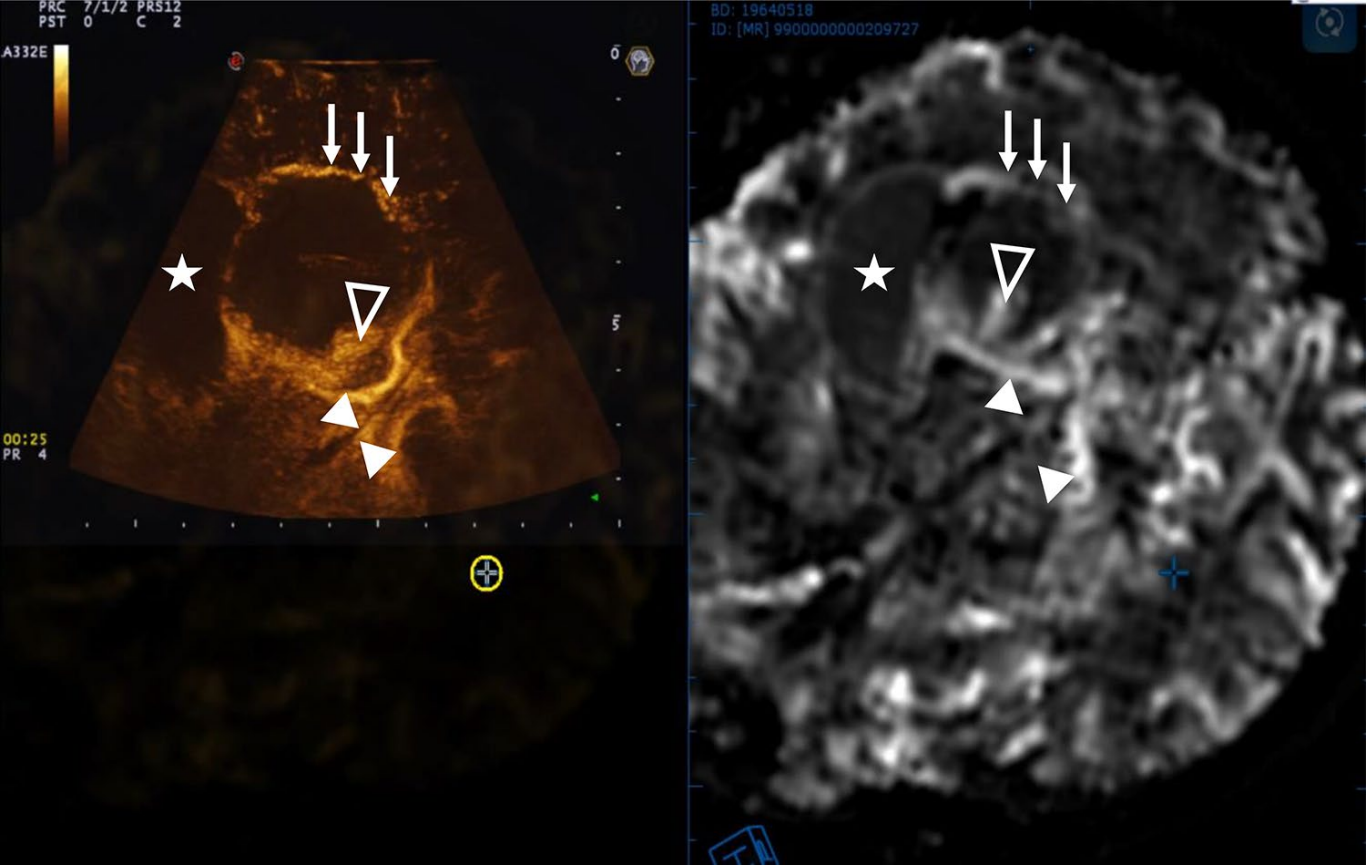
Fusion imaging and navigation between direct cerebral CEUS imaging and MRI perfusion imaging: CEUS image is displayed on the left of the screen coupled with the co-planar MRI on the right side. The two imaging modalities are linked together and the MRI follow the real-time CEUS modality as the US probe is tracked in the 3D space. From a qualitative standpoint MBs distribution grossly follows Gadolinium distribution on MRI as visible with anatomic landmarks: three white arrows, tumor periphery—star, tumor necrotic center—empty arrowhead, tumor bulk—double arrowheads, internal cerebral veins.

Fusion imaging and navigation between direct cerebral CEUS imaging and a non-invasive perfusion imaging method such as perfusion MRI could be exploited in the future to predict MBs cerebral distribution using a non-invasive imaging modality, validating perfusion MR as a biomarker to fully characterize the spatial and temporal distribution of MBs in the healthy and pathological human brain. Such a study is actually running and, as visible in Fig. 7, it is possible to observe, from a qualitative standpoint, a correlation between CEUS and perfusion MR. To this end, replacing the patient’s bone flap after neurosurgical procedures with an acoustic transparent prosthesis will allow to perform CEUS guided visualization of the surgical cavity^44,46,47^ and to assess quantitatively brain tissue perfusion and eventual response to chemotherapy^16^ on the imaging side; from a therapeutic standpoint in the future, we also envisage the possibility to perform MB mediated treatments with direct MB imaging as opposed to indirect visualization or detection^4,48^ and also to assess BBB opening outcome^49^. This findings might also be implemented using novel MB based US imaging techniques such as ultrasound localization microscopy (ULM) which improves both spatial resolution and penetration depth whith increased sensitivity due to the joint use of ultrasound contrast agents and ultrafast imaging, allowing for non-invasive deep microvascular imaging at the microscopic scale^50^. Future clinical applications using MBs for both surgical and/or FUS guidance should consider data obtained with intraoperative CEUS as a strategy for more precisely mapping the accumulation of MBs in regions intended to be targeted. This approach can ultimately allow for better treatment planning, more consistent treatments across patients, and better prediction of surgical resection or clinical responses to FUS BBB opening.

## Supplementary Information

Supplementary Video 1.

Supplementary Video 2.

Supplementary Information 1.

Supplementary Information 2.

## Abbreviations

MB: Microbubble
UCA: Ultrasound contrast agent
CEUS: Contrast enhanced ultrasound
FUS: Focused ultrasound
BBB: Blood brain barrier
GBM: Glioblastoma
HGG: High grade glioma
FOV: Field of view
ROI: Region of interest
TIC: Time intensity curve
AUC: Area under the curve
PE: Peak of enhancement
PCD: Passive cavitation detection
CT: Computed tomography
MR: Magnetic resonance
