# Supplementary material for: Quantitative analysis of in-vivo microbubble distribution in the human brain
Source: Sci Rep. 2021 Jun 3;11:11797. doi: 10.1038/s41598-021-91252-w (PMC8175375; doi:10.1038/s41598-021-91252-w)

**Supplementary Figure**: Graphical User Interface (GUI) developed using MATLAB’s App Designer. Upper panel: video files are loaded into the GUI and it is broken down into the video’s individual frames, which are stored as an array of images.  Once a frame is selected, the user can begin to draw ROIs - circular, rectangular, and freehand drawing options (red and blue circles).

The software calculates an average intensity value for every saved frame, applying the mask to each image. Lower panel: under the analysis tab output graphs can be plotted. These plots show the change in the region’s average intensity over the length of the video and include a line and labeled point depicting the maximum intensity.


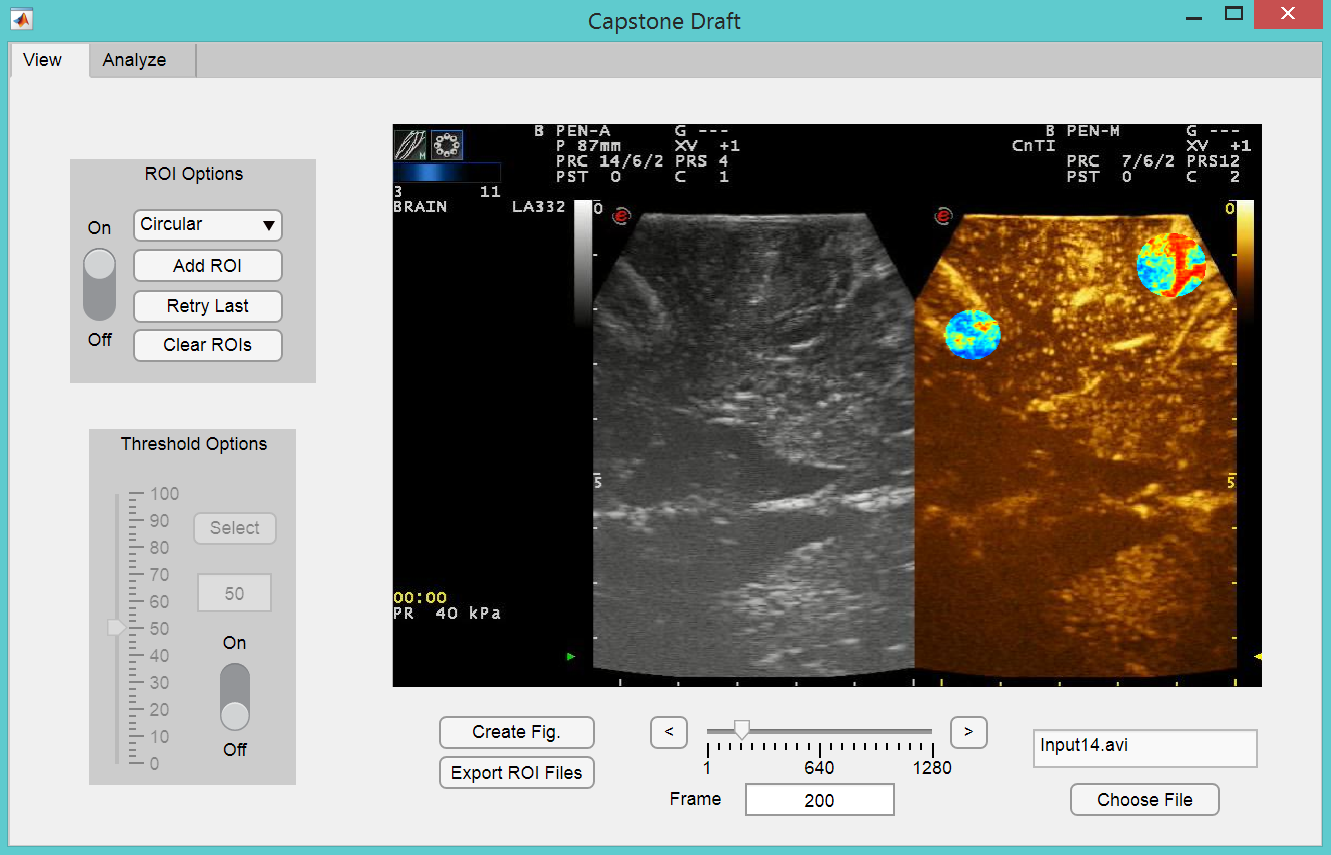


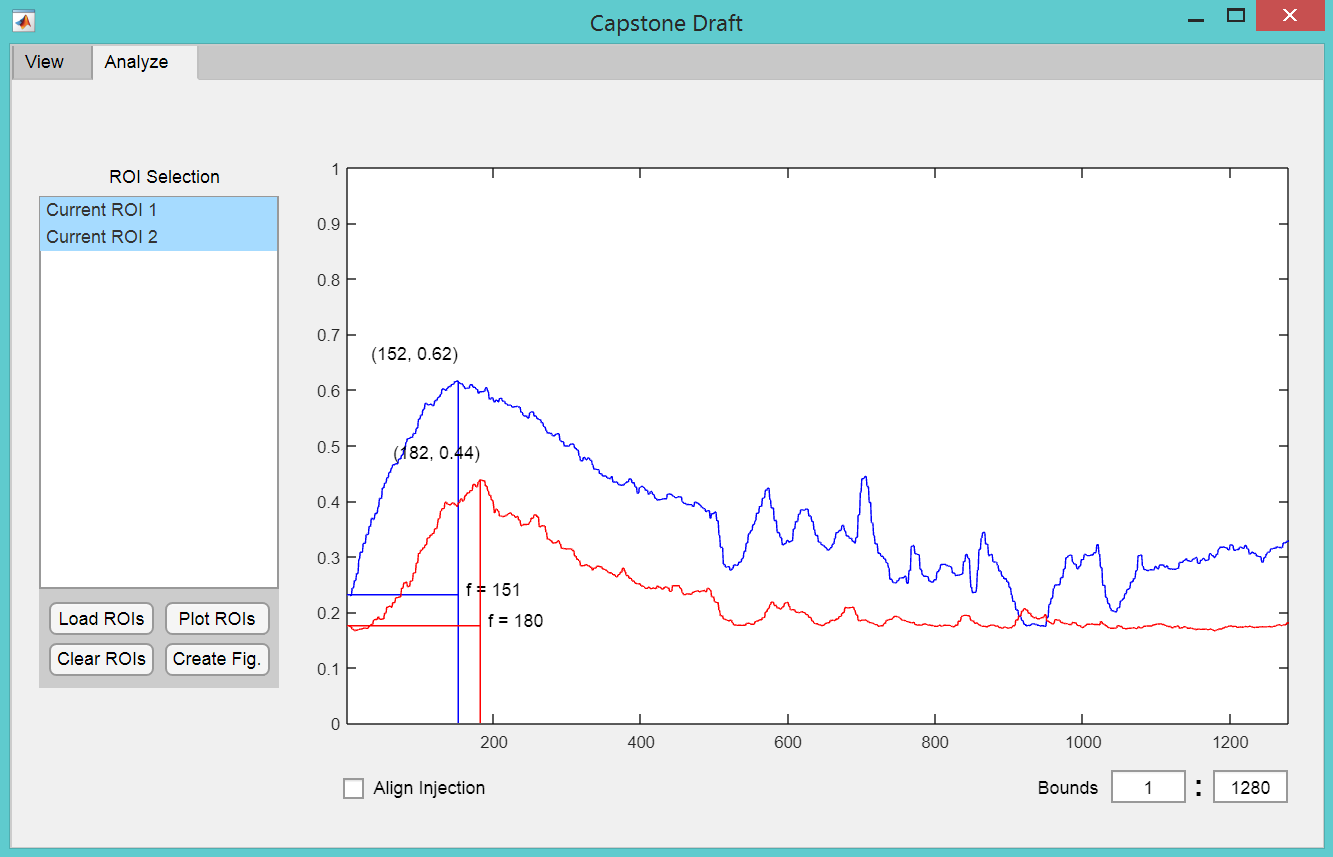

Supplement: Supplementary file 3 — Supplementary Information 1. [file 41598_2021_91252_MOESM3_ESM.docx]
