# Supplementary material for: Quantitative analysis of in-vivo microbubble distribution in the human brain
Source: Sci Rep. 2021 Jun 3;11:11797. doi: 10.1038/s41598-021-91252-w (PMC8175375; doi:10.1038/s41598-021-91252-w)
Supplement: Supplementary file 4 — Supplementary Information 2. [file 41598_2021_91252_MOESM4_ESM.docx]

Supplementary material to: Quantitative Analysis of *In-vivo* Microbubble Distribution in the Human Brain

Francesco Prada^a,b,c^, Antonio G. Gennari^d,e^, Ian M. Linville^f^, Michael E. Mutersbaugh^f^, Zhihang Chen^f^, Natasha Sheybani^f^, Francesco DiMeco^b,g,h^, Frederic Padilla^c,i^, John A. Hossack^f^

Affiliations

^a^ Department of Neurological Surgery, University of Virginia Health System, Charlottesville, VA, USA

^b^ Department of Neurosurgery, Fondazione IRCCS Istituto Neurologico C. Besta, Milan, Italy

^c^ Focused Ultrasound Foundation, Charlottesville, VA, USA

^d^ Neuroradiology Unit, Fondazione IRCCS Istituto Neurologico C. Besta, Milan, Italy

^e^ Department of Radiology, Cattinara Hospital, University of Trieste, Trieste, Italy

^f^ Biomedical Engineering, University of Virginia, Charlottesville, VA, USA

^g^ Department of Pathophysiology and Transplantation, University of Milan, Milan, Italy

^h^ Department of Neurological Surgery, Johns Hopkins Medical School, Baltimore, MD, USA

^i^ Department of Radiology, University of Virginia Health System, Charlottesville, VA, USA


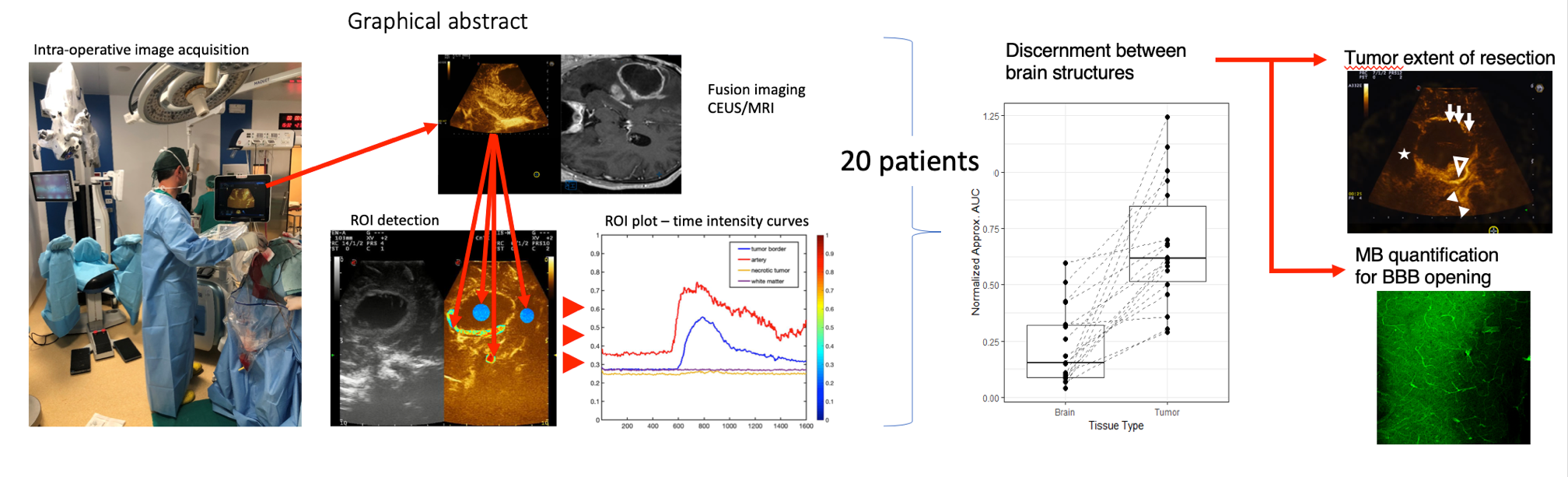


Legend to supplementary material 1: graphical abstract summarizing workflow (from left to right). Contrast enhanced ultrasound (CEUS) videos are acquired intra-operatively during cranial neurosurgical procedures for brain tumor removal through the craniotomic window by the first author (FP). CEUS videos of adequate length (< 40 sec) were selected. An offline quantitative analysis is then performed using a dedicated software, adding region of interest on selected structures – at least an artery as a reference, brain parenchyma and tumor bulk – providing time intensity curves. Time intensity curves (TIC) were then analyzed (boxplot) showing a consistent correlation in terms of microbubble (MBs) perfusion between different anatomical strucutures across all patients. Lastly future clinical applications of our findings are highlighted, namely quantitative data for intra-operative surgical guidance an MBs perfusion analysis to plan MB mediated treatment. radiolo


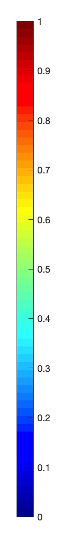

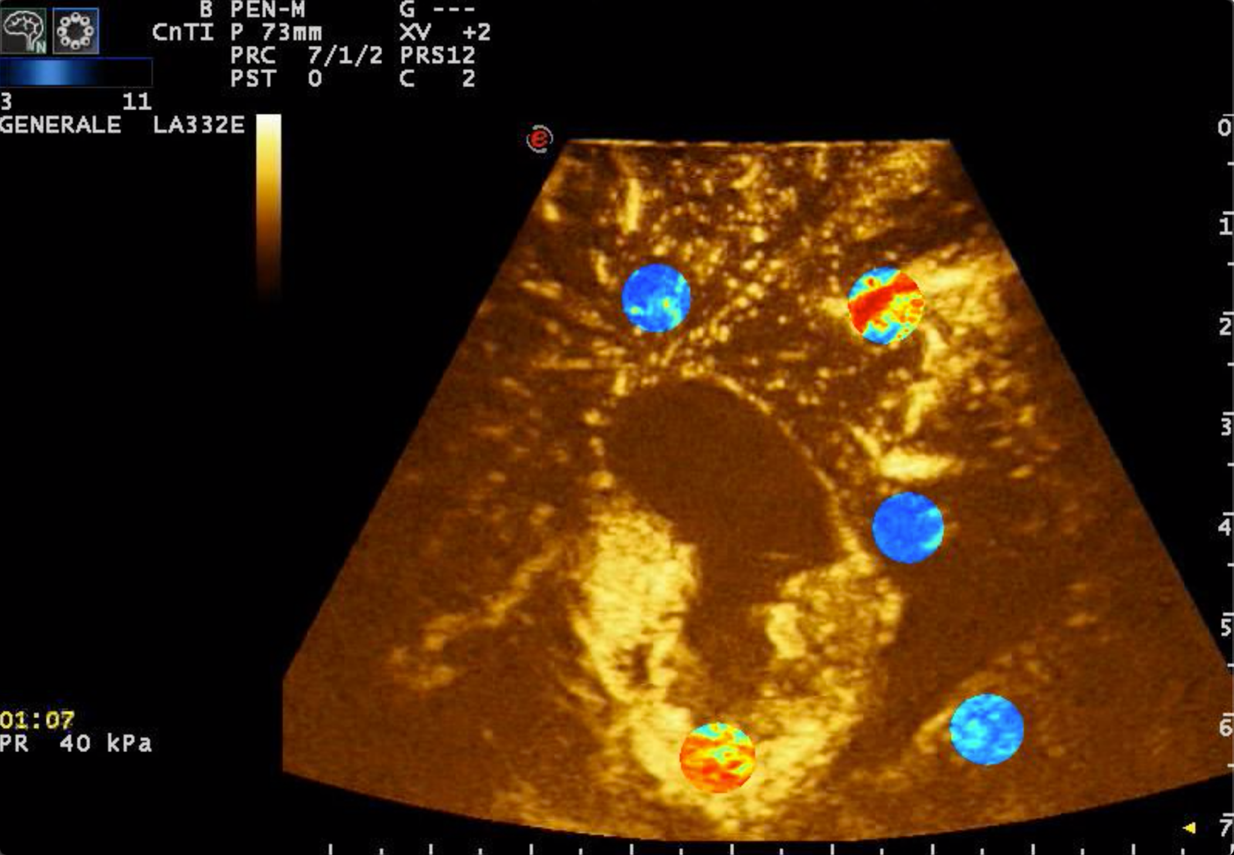


White matter

Sulcal artery

Corpus callosum

Caudate nucleus

Tumor

Legend to supplementary material 2: intra-operative CEUS screenshot in a case of a thalamic GBM acquired in a coronal section through a frontal craniotomy. Circular ROIs are superimposed to the video highlighting MBs’ concentration in time in different structures (periventricular white matter, sulcal artery, corpus callosum, caudate nucleus, tumor) using a colorimetric scale.


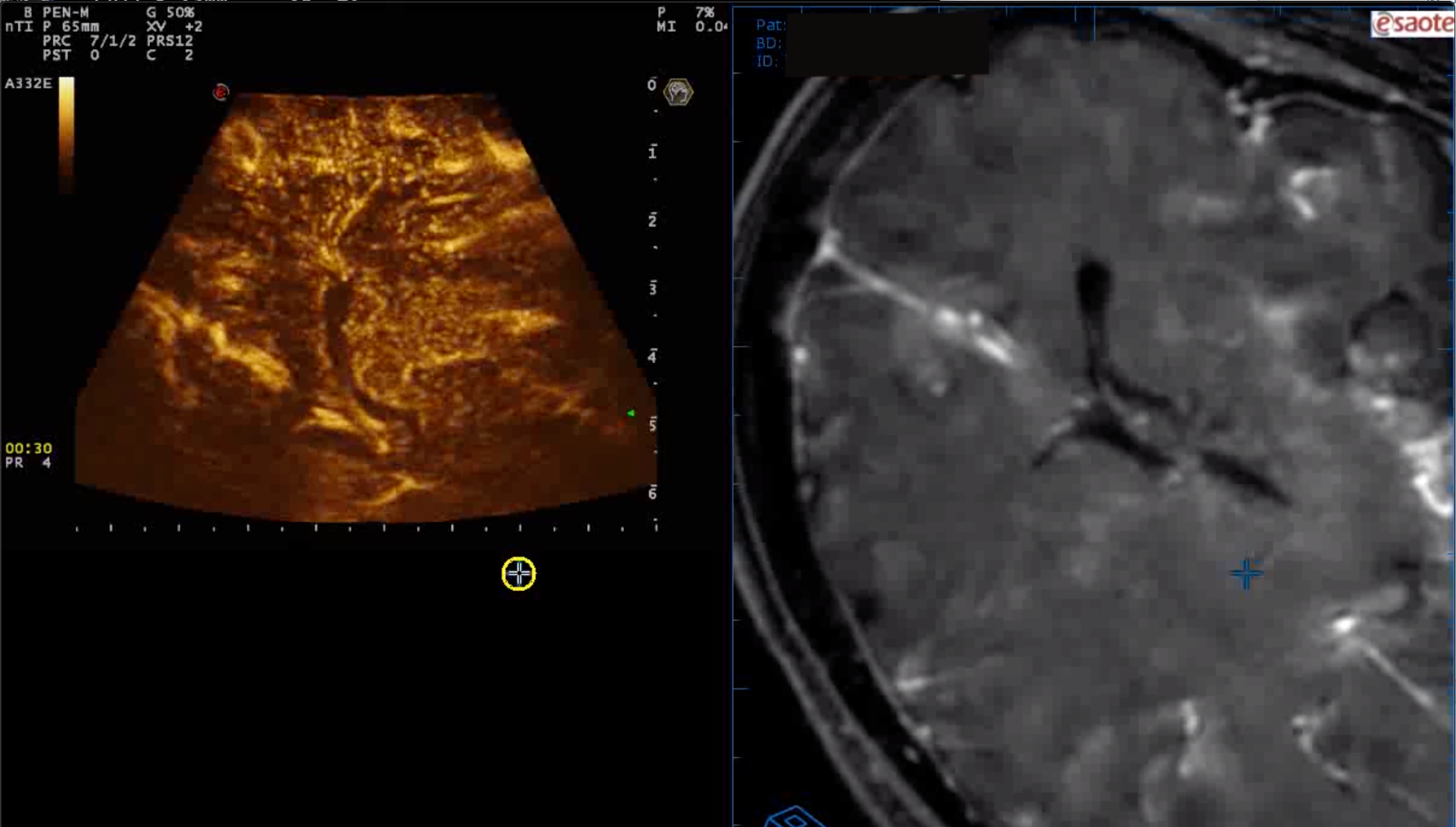


Legend to supplementary material 3: intra-operative video using fusion imaging between real-time CEUS (left) and pre-operative dynamic contrast enhance magnetic resonance imaging. (DCE-MRI) in a case of tuberculum sellae meningioma through a pterional craniotomic window. The two imaging modalities are co-planar, thus showing the exact same cut, allowing for direct comparison. This will permit to translate real-time intra-operative data to pre-operative imaging for planning.
